# Impact and effectiveness of Rotavin-M1 under conditions of routine use in two provinces in Vietnam, 2016–2021, an observational and case–control study

**DOI:** 10.1016/j.lanwpc.2023.100789

**Published:** 2023-05-18

**Authors:** Nguyen Van Trang, Jacqueline E. Tate, Le Thi Phuong Mai, Thiem Dinh Vu, Nguyen Tu Quyet, Ly Khanh Thi Le, Mai Ngoc Thi Chu, Mai Phuong Ngoc Tran, Thao Phuong Thi Pham, Huong Thuy Nguyen, Nguyen Dang Hien, Baoming Jiang, Catherine Yen, Duong Nhu Tran, Dang Duc Anh, Umesh D. Parashar, Rotavin VE team, Lai Tuan Anh, Vu Duc Thanh, Le Van Sanh, Dang Thi Dieu Thuy, Dinh Cong Trang, Nguyen Quoc Phong, Doan Hong Truong, Tran Van Tai, Phạm Van Dung, Do Van Van

**Affiliations:** a National Institute of Hygiene and Epidemiology, Hanoi, Viet Nam; b United States Centers for Disease Control and Prevention, Atlanta, GA, USA; c Center for Production and Development of Vaccines and Biologicals, Hanoi, Viet Nam

**Keywords:** Rotavirus, Rotavirus vaccine, Vaccine effectiveness, Vietnam

## Abstract

**Background:**

Half of diarrhea hospitalizations in children aged <5 years in Vietnam are due to rotavirus. Following introduction of a locally developed and licensed oral rotavirus vaccine, Rotavin-M1, into the routine immunization program in two Vietnamese provinces, Nam Dinh and TT Hue, we describe changes in rotavirus positivity among children hospitalized for diarrhea and calculate vaccine effectiveness against moderate-to-severe rotavirus hospitalizations.

**Methods:**

Active rotavirus surveillance among children <5 years began in December 2016 at sentinel hospitals in districts where rotavirus vaccine was introduced in December 2017. To estimate reductions in rotavirus detection, we calculated risk ratios comparing rotavirus positivity pre- and post-vaccine introduction. We used a test-negative case–control design to calculate vaccine effectiveness.

**Findings:**

From December 2016 to May 2021, 7228 children <5 years hospitalized for diarrhea were enrolled. Following introduction, Rotavin-M1 coverage was 77% (1066/1377) in Nam Dinh and 42% (203/489) in TT Hue. In Nam Dinh, rotavirus positivity among children <5 years significantly declined by 40.6% (95% CI: 34.8%–45.8%) during the three-year post-vaccine introduction period. In TT Hue, no change in rotavirus positivity was observed. Among children aged 6–23 months, a 2-dose series of Rotavin-M1 was 57% (95% CI: 39%–70%) effective against moderate-to-severe rotavirus hospitalizations.

**Interpretation:**

Higher vaccination coverage in Nam Dinh than TT Hue likely contributed to substantial declines in rotavirus positivity observed in Nam Dinh following rotavirus vaccine introduction. Robust vaccine effectiveness was observed through the second year of life. National rotavirus vaccine introduction with high coverage may have substantial impact on reducing rotavirus disease burden in Vietnam.

**Funding:**

10.13039/100000865Bill and Melinda Gates Foundation.

**Research in context**

**Evidence before this study**

We searched PubMed for articles published in any language up to October 2022 using the search terms “(rotavirus vaccine) AND (“Viet nam” OR Vietnam)”. We identified 58 articles, of which 3 contained data from clinical trials of Rotavin-M1, 3 contained data from clinical trials of RotaTeq in Vietnam, 1 contained data from a clinical trial of Rotarix in Vietnam, and 1 contained data from a clinical trial of Rotavac in Vietnam. The clinical trial for RotaTeq was the only trial identified with data on a clinical endpoint and this vaccine was 63.9% effective against severe rotavirus gastroenteritis in Vietnam. The clinical trials for all other rotavirus vaccines including Rotavin-M1 had immunogenicity, reactogenicity, and/or safety endpoints. One article examined effectiveness of rotavirus vaccines received on the private market in southern Vietnam and found an effectiveness of 69.7% with most children having received Rotarix. This study also found that rotavirus positivity among children <5 years of age declined from 55% in 2013 to 44% in 2018 with only 4% of enrolled children having received at least one dose of rotavirus vaccine.

**Added value of this study**

In our evaluation, Rotavin-M1 was administered through the routine expanded program on immunization system with all children living in the selected districts eligible to receive the vaccine. Children received rotavirus vaccine during the same visit as other routinely recommended vaccines. The vaccine was 57% effective against hospitalization for moderate-to-severe rotavirus disease with significant reductions in rotavirus positivity among children hospitalized for diarrhea observed when high vaccination coverage was achieved.

**Implications of all the available evidence**

National rotavirus vaccine introduction with high coverage may have substantial impact on rotavirus disease burden in Vietnam. Continued surveillance to monitor the impact and effectiveness following the national introduction of rotavirus vaccine in Vietnam is important.

## Introduction

Despite the availability of safe and effective vaccines, rotavirus persists as one of the most common causes of severe gastroenteritis in children <5 years of age worldwide.^1^^,^^2^ Since 2009, the World Health Organization (WHO) has recommended the use of rotavirus vaccines in all countries,^3^ and four rotavirus vaccines have been pre-qualified by WHO for use globally.^4^ By October 2022, >110 countries had introduced rotavirus vaccines into their routine immunization programs.^5^ The impact of rotavirus vaccines on disease burden has been widely documented with some of the largest absolute declines in pediatric rotavirus hospitalizations observed in countries with the highest disease burden.^6^^,^^7^

Since 1998, Vietnam has generated timely and geographically-representative surveillance data on severe rotavirus disease in Vietnamese children using standardized protocols for enrollment and diagnostic evaluation.^8^^,^^9^ Rotavirus accounts for almost half of all diarrhea hospitalizations among children <5 years of age in Vietnam with most children presenting with acute watery diarrhea between 6 and 23 months of age.^8^^,^^9^ In a large two-site birth cohort study, over half of the samples that had an etiology identified were positive for rotavirus and 13% of all rotavirus episodes were hospitalized.^10^ Given this substantial disease burden and an insecure global supply of rotavirus vaccines, the Government of Vietnam encouraged local rotavirus vaccine development and production so that the country could be self-reliant on an affordable vaccine. The Centre for Research and Production of Vaccines and Biologicals of Vietnam's Ministry of Health developed a liquid-frozen, oral, live attenuated monovalent rotavirus vaccine, Rotavin-M1, which contains ≥2 × 10^6^ FFU of strain G1P[8] per 2 mL dose.^11^ This vaccine was licensed for local use in May 2012 and currently is available on the private market. Although safety and immunogenicity data for Rotavin-M1 were sufficient for local licensure,^12^ clinical efficacy and effectiveness data for this vaccine are not available.

To evaluate vaccine impact and effectiveness under conditions of routine use, Rotavin-M1 was introduced through the Expanded Programme on Immunization in selected districts of two provinces, Nam Dinh and Thua Thien Hue (TT Hue), as part of a demonstration project. Active rotavirus surveillance was established at hospitals serving children residing in these districts. The objective of this manuscript is to describe trends in rotavirus hospitalizations among children <5 years of age pre- and post-rotavirus vaccine introduction and vaccine effectiveness against hospitalization for moderate-to-severe rotavirus disease.

## Methods

### Rotavirus vaccine introduction

Rotavin-M1 was introduced in December 2017 into the routine immunization program in selected districts in Nam Dinh and TT Hue provinces and continued through December 2020. Prior to introduction, training sessions on Rotavin-M1 immunization were conducted from July to August 2017 in all participating hospitals in Nam Dinh and TT Hue provinces for community health center staff. While Rotavin-M1 can be stored at 2–8 °C for up to 2 months, it must be kept at −20 °C for longer periods. Thus, the vaccine was stored and maintained at least at −20 °C during shipping to provinces and districts. The vaccine was then transferred to community health centers on the day of vaccination and kept at 4 °C. Two doses of vaccine were recommended for infants 2 and 3 months of age and were administered at the same time as other routine infant immunizations. To ensure timely administration of rotavirus vaccine, children had to receive the first dose of vaccine before 14 weeks of age and the last dose before 6 months of age. Children who presented at 14 weeks of age or older for their first immunization visit were not vaccinated against rotavirus. Rotavirus vaccine administration information was recorded in the vaccination logbook maintained at the community health center and in the electronic vaccination registry. A child was considered vaccinated against rotavirus if dates of rotavirus vaccine administration were recorded in the vaccination logbook, the electronic vaccination registry, and/or the parent-maintained vaccine card. If dates of vaccination for other routine immunizations were available in one of these sources but date of rotavirus vaccination was not, the child was considered unvaccinated against rotavirus. Children whose parents reported that they had been vaccinated but no record of vaccination could be found in any of these sources and children with unknown vaccination status were excluded from the vaccine effectiveness analysis.

### Active surveillance for rotavirus hospitalizations

Active surveillance for rotavirus hospitalizations was conducted in 4 district hospitals (Giao Thuy District Hospital, Hai Hau District Hospital, Truc Ninh District Hospital, and Xuan Truong District Hospital) in Nam Dinh and 2 district hospitals (Huong Tra District Hospital, Phu Vang District Hospital) and 1 tertiary hospital (Central Hue Hospital) in TT Hue. Surveillance started in all hospitals in December 2016 and stopped at Central Hue Hospital in October 2018 and at all other hospitals in May 2021. Children were eligible for enrollment if they were admitted to a surveillance hospital with acute gastroenteritis defined as 3 or more looser-than-normal stools within a 24h period with duration less than 14 days prior to admission. Demographic and clinical data were collected from parental interview and from medical records using a standardized case report form. Vaccine information was collected from the vaccination logbook maintained at the community health center and from the electronic vaccination registry as described above. A stool specimen was collected within 48 h of admission, kept at −20 °C at district hospitals and monthly shipped to central laboratory at NIHE, aliquoted and kept at −80 °C until testing.

### Rotavirus detection

Stool specimens were shipped frozen to the National Institute of Hygiene and Epidemiology where they were tested for rotavirus by a commercial enzyme immunoassay according to the Premier Rotaclone kit manufacturer's instruction (Meridian Premier™, Ohio, USA).

Viral RNA was extracted from rotavirus-positive samples using a QIAamp Viral RNA Mini Kit or automatic extraction system QIAcube HT using extraction kit Cador Pathogen 96 Qiacube HT Kit according to the manufacturer's instructions (Qiagen, Hilden, Germany). For G genotyping, the primers used were described by Fujii et al.,^13^ primers for P genotyping were used according to Esona et al.^14^ The VP7 and VP4 genes were amplified by reverse transcription polymerase chain reaction (RT-PCR) amplification using Qiagen Onestep RT-PCR (Qiagen, Hilden, Germany). The amplification condition was: 50 °C for 30 min, 95 °C for 15 min, and followed by 40 cycles of 94 °C for 1min (VP7)/30s (VP4), 50 °C for 30 s and 72 °C for 1 min (VP7)/45s (VP4) with a final extension step of 72 °C for 10 min. PCR products obtained were identified by 2% agarose gel electrophoresis, and the amplified products meeting the target segment size were judged as positive.

### Analyses

Rotavirus seasonality differed in Nam Dinh and TT Hue provinces. In Nam Dinh, where distinct seasonal peaks in rotavirus were observed, annual rotavirus seasons were defined from April to March with the pre-vaccine period defined as April 2017–March 2018 and three post-vaccine introduction annual rotavirus seasons defined as April 2018–March 2019, April 2019–March 2020, and April 2020–March 2021. In TT Hue, where less seasonality during the year was seen, annual rotavirus seasons were defined from January to December with the pre-vaccine period defined as January 2017–December 2017 and three post-vaccine introduction annual rotavirus seasons defined as January 2018–December 2018, January 2019–December 2019, and January 2020–December 2020. Diarrhea severity was classified using the 20-point Veskari score assessed at the time of admission.^15^ Moderate-to-severe diarrhea was defined as a Vesikari score ≥11.

We assessed rotavirus vaccine impact on the burden of disease in several different ways. First, we visually examined trends over time by plotting rotavirus positive and negative hospitalizations and rotavirus vaccination coverage over time using a three-month moving average. As rotavirus vaccination does not prevent other causes of diarrhea, vaccination coverage in the population was estimated using children with confirmed vaccination status that were hospitalized for non-rotavirus diarrhea. Second, to estimate reductions and associated 95% confidence intervals (CI) in positivity by year, we used (1-risk ratio)∗100, with the risk ratio comparing the pre- and post-vaccine introduction positivity. Third, the median age at rotavirus hospitalization in the pre- and post-vaccine introduction periods were compared using the Wilcoxon rank sum test. Fourth, the Mantel–Haenszel test was used to assess trends in severity over time. Central Hue Hospital was excluded from all impact analyses as surveillance ended in this site during October 2018.

To calculate vaccine effectiveness among children 6–23 months of age, we used a test-negative case–control design and restricted to children who were age-eligible to receive rotavirus vaccine. A child was age-eligible to receive the vaccine if born less than 14 weeks before the start of the vaccination program and was 6 or more weeks of age before the end of the vaccination program. Cases were children with acute gastroenteritis who tested positive for rotavirus and controls were children with acute gastroenteritis who tested negative for rotavirus. Sociodemographic and clinical characteristics of children with acute gastroenteritis who tested positive and negative for rotavirus were compared using the Mantel–Haenszel chi-square test. Rotavirus vaccination status was confirmed as described above. A dose of rotavirus vaccine was considered relevant if it was administered at least 14 days prior to admission. Vaccine effectiveness was calculated as (1-odds ratio)∗100, where the odds ratio is the adjusted odds ratio for the rotavirus vaccination rate among cases compared with controls. The odds ratio was calculated using unconditional logistic regression. Potential confounders were defined as any variable that changed the odds ratio by >10%. To assess a VE of 60%, we estimated that a minimum of 167 cases with rotavirus diarrhea were required with a control-to-case ratio of 2:1 and an expected vaccine coverage of 70% among controls. Vaccine effectiveness estimates were calculated for children who received the full two dose vaccine series compared with unvaccinated children.

All analyses were conducted using SAS version 9.4 (Cary, NC).

### Role of the funding source

Funding for this project was provided by the Bill & Melinda Gates Foundation, which had no role in the study design or in the collection, analysis, or interpretation of the data.

## Results

From December 2016 to May 2021, 7228 children <5 years of age hospitalized for diarrhea were enrolled (4662 children in Nam Dinh and 2566 children in TT Hue), and 6777 (94%) had a stool specimen collected (4428 (95%) in Nam Dinh and 2349 (92%) in TT Hue). Of the 6626 specimens tested, 2164 (32%) were positive for rotavirus (1553 (35%) positive in Nam Dinh and 611 (26%) positive in TT Hue). Among 1377 children in Nam Dinh and 489 children in TT Hue age-eligible to receive rotavirus vaccine that were hospitalized for non-rotavirus diarrhea and had a verified vaccination status, coverage with ≥1 dose of Rotavin-M1 in Nam Dinh was 77% (n = 1066) and 42% (n = 203) in TT Hue (Supplemental Table S1).

### Vaccine impact in Nam Dinh

**Fig. 1:**
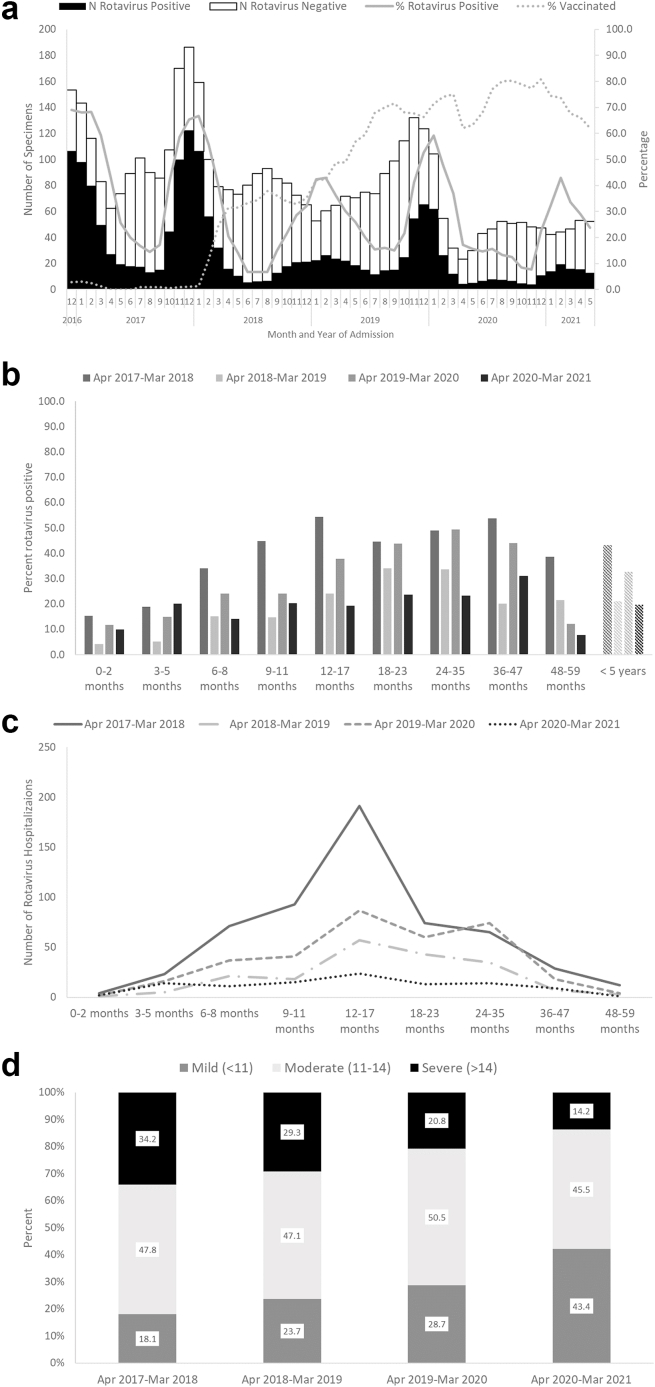
a) Number of rotavirus positive and negative children <5 years of age hospitalized for rotavirus diarrhea, proportion rotavirus positive and proportion vaccinated, b) Rotavirus positivity by age and rotavirus season, c) Number of rotavirus hospitalizations by age and rotavirus season, and d) distribution of diarrhea severity by season, Nam Dinh Province, December 2016–May 2021.

In Nam Dinh, rotavirus hospitalizations among children <5 years of age exhibited a clear seasonal trend prior to vaccine introduction with a sharp peak from October to March (Fig. 1a). Following vaccine introduction, the peaks became blunted with an alternating pattern of low activity in the first full year following vaccine introduction and a moderate increase in activity the subsequent year but still below baseline activity. In March 2020 at the start of the global COVID-19 pandemic, there was a sharp decline in all-cause diarrhea hospitalizations that persisted until the end of the surveillance period.

Similar patterns were observed across the age spectrum. The proportion of diarrhea hospitalizations due to rotavirus decreased in all age groups in the year following rotavirus vaccine introduction but increased the following year especially among children too old to receive the vaccine (Fig. 1b) resulting an uptick in rotavirus positivity in the second post-vaccine introduction year. The pattern was less clear in the youngest (<6 months) and oldest (≥48 months) age groups where the number of children enrolled was low.

Overall among children <5 years of age, rotavirus positivity significantly declined by 40.6% (95% CI: 34.8%–45.8%) over the three year post-vaccine introduction period with larger declines in the first (risk reduction (RR): 51.0%; 95% CI: 43.6%–57.5%) and third (RR: 54.5%; 95% CI: 45.3%–62.1%) years post-vaccine introduction than in the second year post-vaccine introduction (RR: 24.5%; 95% CI: 15.9%–32.2%) (Table 1). Reductions in rotavirus positivity were most pronounced in children <1 year of age and least pronounced in children 2–4 years of age. Coverage with ≥1 dose of Rotavin-M1 among all children <5 years of age increased from 35.7% in the first year post-vaccine introduction to 75.0% in the third year post-vaccine introduction with the highest coverage achieved in the <1 year and 1 year age groups which were fully age-eligible to be vaccinated by the end of the surveillance period.

**Table 1:** Rotavirus positivity, risk reduction, and rotavirus vaccine coverage pre- and post-rotavirus vaccine introduction in Nam Dinh Province, April 2017–March 2021 and Hue Province, January 2017–December 2020.

	<5 years			<1 year			1 year			2–4 years		
	Rotavirus positivity	Risk reduction	Rotavirus vaccination coverage^c^	Rotavirus positivity	Risk reduction	Rotavirus vaccination coverage^c^	Rotavirus positivity	Risk reduction	Rotavirus vaccination coverage^c^	Rotavirus positivity	Risk reduction	Rotavirus vaccination coverage^c^
	n/N (%)	RR (95% CI)	n/N (%)	n/N (%)	RR (95% CI)	n/N (%)	n/N (%)	RR (95% CI)	n/N (%)	n/N (%)	RR (95% CI)	n/N (%)
**Nam Dinh province**												
Pre-vaccine Apr 2017–Mar 2018^a^	562/1301 (43.2%)	ref	18/670 (2.7%)	191/565 (33.8%)	ref	17/362 (4.7%)	265/518 (51.2%)	ref	1/228 (0.4%)	106/218 (48.6%)	ref	0/80 (0.0%)
Year 1 post-vaccine Apr 2018–Mar 2019	190/898 (21.2%)	51.0% (43.6%, 57.5%)	243/680 (35.7%)	45/382 (11.8%)	65.2% (53.1%, 74.1%)	212/332 (63.9%)	100/363 (27.5%)	46.2% (35.1%, 55.3%)	29/248 (11.7%)	45/153 (26.9%)	39.5% (19.9%, 54.3%)	2/100 (2.0%)
Year 2 post-vaccine Apr 2019-Mar 2020	339/1039 (32.6%)	24.5% (15.9%, 32.2%)	455/681 (66.8%)	96/448 (21.4%)	36.6% (21.7%, 48.7%)	280/347 (80.7%)	147/367 (40.1%)	21.7% (9.0%, 32.7%)	166/214 (77.6%)	96/224 (42.9%)	11.9% (−8.1%, 28.1%)	9/120 (7.5%)
Year 3^b^ post-vaccine Apr 2020–Mar 2021	103/524 (19.7%)	54.5% (45.3%, 62.1%)	315/420 (75.0%)	42/242 (17.4%)	48.7% (30.8%, 61.9%)	155/200 (77.5%)	37/180 (20.6%)	59.8% (45.8%, 70.2%)	122/143 (85.3%)	24/102 (23.5%)	51.6% (29.6%, 66.8%)	38/77 (49.4%)
Year 1 and 2 post-vaccine Apr 2018–Mar 2019	529/1937 (27.3%)	36.8% (30.4%, 42.5%)	698/1361 (51.3%)	141/830 (17.0%)	49.7% (39.3%, 58.4%)	492/679 (72.5%)	247/730 (33.8%)	33.8% (24.5%, 42.0%)	195/462 (42.2%)	141/377 (37.4%)	23.1% (7.1%, 36.3%)	11/220 (5.0%)
Total post-vaccine Apr 2018–Mar 2021	632/2461 (25.7%)	40.6% (34.8%, 45.8%)	1013/1781 (56.9%)	183/1072 (17.1%)	49.5% (39.8%, 57.6%)	647/879 (73.6%)	284/910 (31.2%)	39.0% (30.7%, 46.3%)	317/605 (52.4%)	165/479 (34.4%)	29.2% (14.8%, 41.1%)	49/297 (16.5%)
**Hue Province**												
Pre-vaccine Jan 2017–Dec 2017^a^	92/453 (20.3%)	ref	2/320 (0.6%)	32/211 (15.2%)	ref	2/164 (1.2%)	30/115 (26.1%)	ref	0/83 (0.0%)	30/127 (23.6%)	ref	0/73 (0.0%)
Year 1 post-vaccine Jan 2018–Dec 2018	129/649 (19.9%)	2.1% (−24.3%, 22.9%)	73/433 (16.9%)	44/268 (16.4%)	−8.3% (−64.4%, 28.7%)	71/219 (32.4%)	50/214 (23.4%)	10.4% (−32.5%, 39.5%)	2/138 (1.4%)	35/167 (21.0%)	11.3% (−36.3%, 42.3%)	0/76 (0.0%)
Year 2 post-vaccine Jan 2019-Dec 2019	62/279 (22.2%)	−9.4% (−45.6%, 17.7%)	80/191 (41.9%)	18/126 (14.3%)	5.8% (−60.6%, 44.8%)	51/106 (48.1%)	25/89 (28.1%)	−7.7% (−69.4%, 31.5%)	29/60 (48.3%)	19/64 (29.7%)	−25.7% (−105.1%, 23.0%)	0/25 (0.0%)
Year 3^b^ post-vaccine Jan 2020–Dec 2020	41/143 (28.7%)	−41.2% (−93.7%, −3.1%)	49/99 (49.5%)	22/90 (24.4%)	−61.2% (−158.9%, 0.6%)	39/67 (58.2%)	13/36 (36.1%)	−38.4% (−135.7%, 18.7%)	8/23 (34.8%)	6/17 (35.3%)	−49.4% (−205.6%, 27.0%)	2/9 (22.2%)
Year 1 and 2 post-vaccine Jan 2018–Dec 2019	191/928 (20.6%)	−1.3% (−26.5%, 18.8%)	153/624 (24.5%)	62/394 (15.7%)	−3.8% (−53.6%, 29.9%)	122/325 (37.5%)	75/303 (24.8%)	5.1% (−36.7%, 34.1%)	31/198 (15.7%)	54/231 (23.4%)	1.0% (−46.1%, 33.0%)	0/101 (0.0%)
Total post-vaccine Jan 2018–Dec 2020	232/1071 (21.6%)	−6.7% (−32.3%, 14.0%)	202/723 (27.9%)	84/484 (17.4%)	−14.4% (−66.3%, 21.2%)	161/392 (41.1%)	88/339 (26.0%)	0.5% (−42.1%, 30.3%)	39/221 (17.6%)	60/248 (24.2%)	−2.4% (−50.2%, 30.1%)	2/110 (1.8%)

a Vaccine introduced late December 2017; January–March 2018 excluded from post-vaccine period due to low vaccination coverage.

b Healthcare visits drastically reduced during this period due to global COVID-19 pandemic.

c Vaccination coverage with at least one dose of Rotavin-M1 was calculated using children age-eligible to receive rotavirus vaccine that were hospitalized for non-rotavirus diarrhea.

Prior to vaccine introduction, rotavirus hospitalizations peaked in children 12–17 months of age (Fig. 1c). The peak in children 12–17 months of age was less pronounced in the post-vaccine introduction period. The median age of children hospitalized for rotavirus increased from 13 months of age (interquartile range (IQR): 10–20 months) in the pre-vaccine period to 17 months (IQR: 12–22 months; p = 0.001) in the first year post-vaccine introduction and to 16 months (IQR: 11–25 months; p = 0.001) in the second year post-vaccine introduction as younger children were protected through vaccination. In the third year post-vaccine introduction during the global COVID-19 pandemic, the median age was 14 months (IQR: 8–23 months; p = 0.65) but overall admissions for rotavirus were low. Severity of diarrhea among enrolled children significantly decreased over the surveillance period from 81.9% with moderate-to-severe diarrhea (Vesikari score ≥11) in the pre-vaccine period to 76.4%, 71.3%, and 59.7% in the first, second, and third seasons post-vaccine introduction respectively (p < 0.001) (Fig. 1d). Similar trends of decreasing severity were seen for children hospitalized with rotavirus positive (p < 0.001) and rotavirus negative (p < 0.001) diarrhea with higher overall severity among rotavirus positive compared with rotavirus negative children (Supplemental Figure S1).

**Fig. 2:**
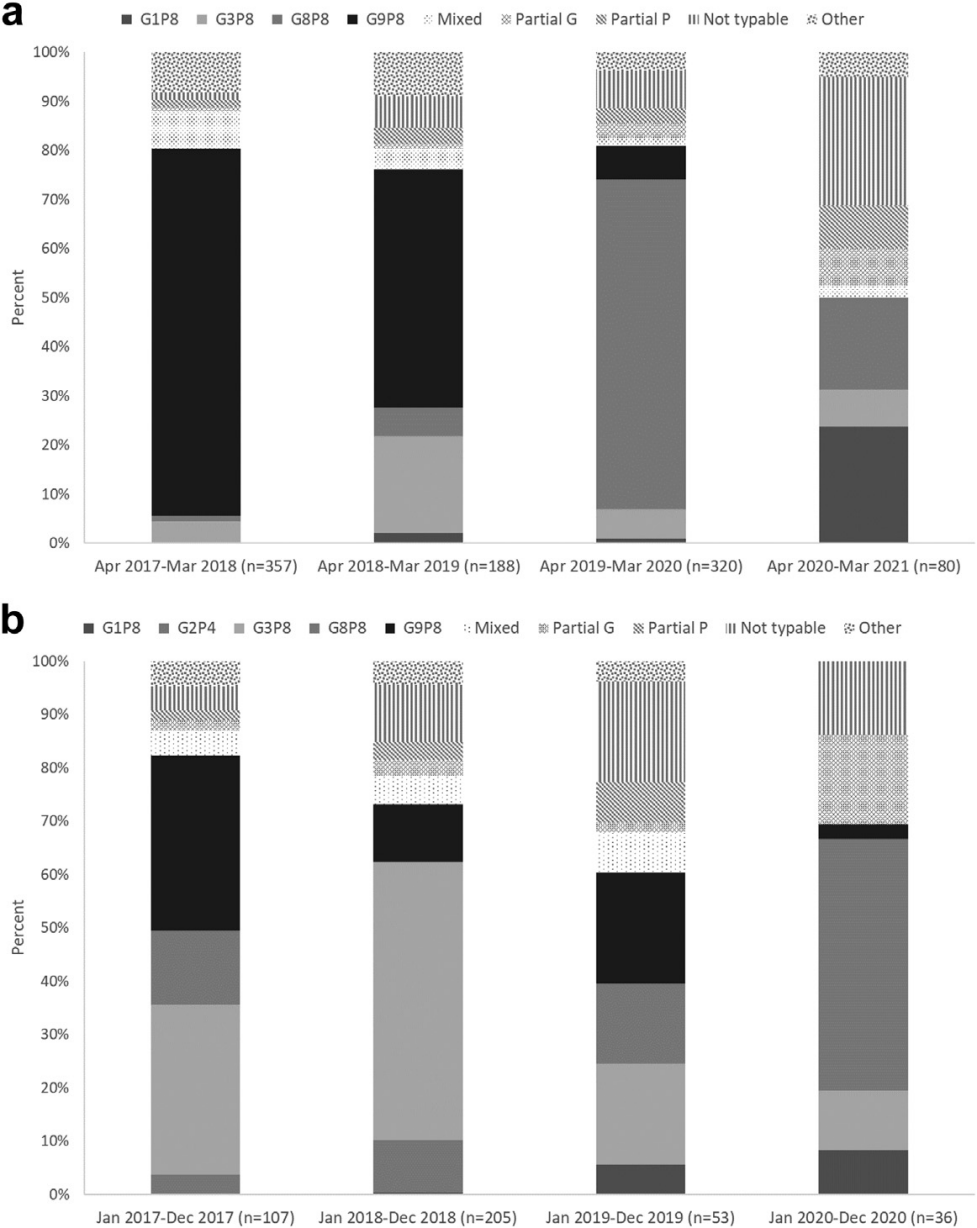
Distribution of circulating rotavirus genotypes among children <5 years of age by rotavirus season in a) Nam Dinh Province, April 2017–March 2021 and b) TT Hue Province, January 2017–December 2020.

The predominating circulating genotypes changed from season to season (Fig. 2a; Supplemental Table S2). In the pre-vaccine season from April 2017 to March 2018, the predominating circulating genotype was G9P[8] (74.8%). In the first post-vaccine season (April 2018–March 2019), G9P[8] (48.4%) and G3P[8] (19.7%) were the most common and G8P[8] (67.2%) predominated in the second post-vaccine season (April 2019–March 2020). In the third post-vaccine season (April 2020–March 2021), G1P[8] (23.8%) and G8P[8] (18.8%) were the most common genotypes although 26.3% of rotavirus positive specimens could not be genotyped and another 16.3% were only partially genotyped.

### Vaccine impact in TT Hue

**Fig. 3:**
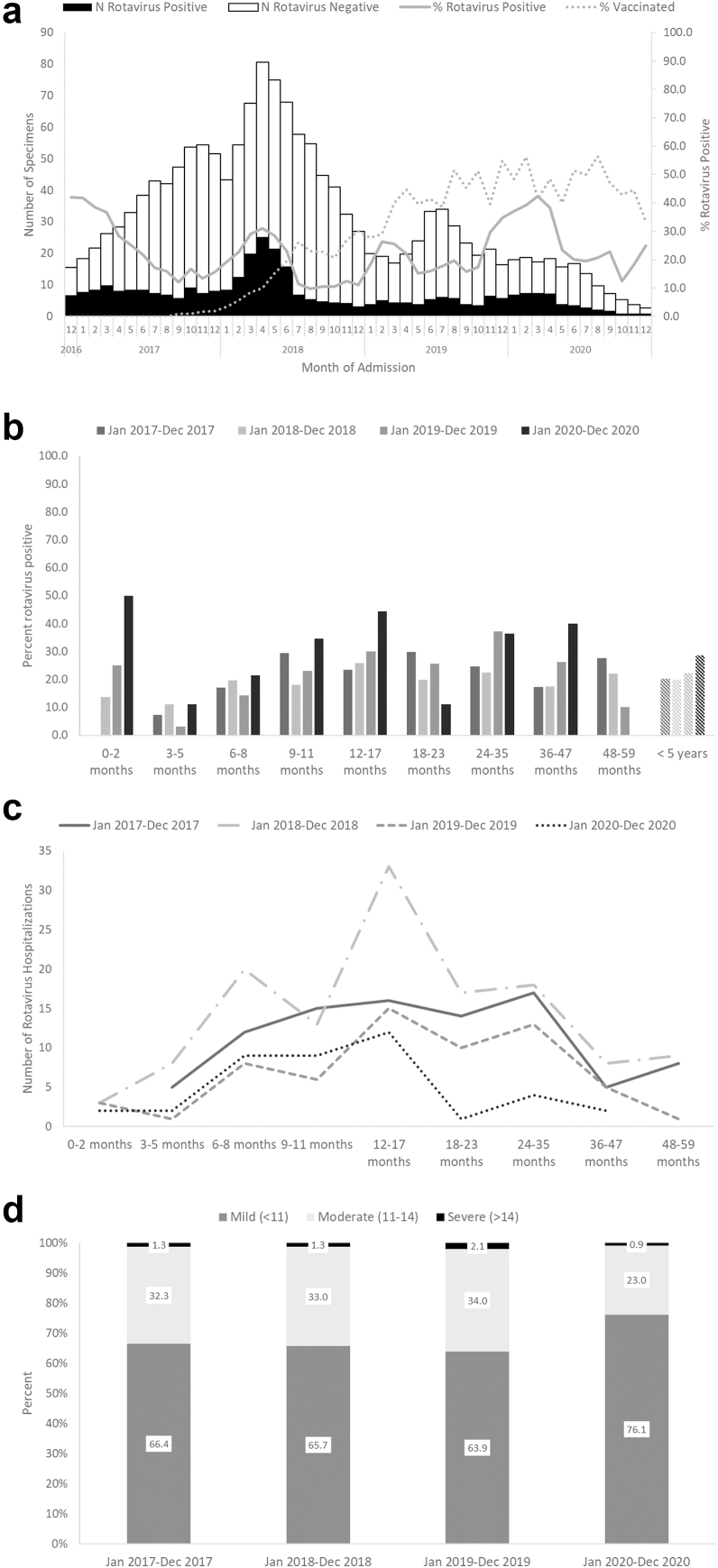
a) Number of rotavirus positive and negative children <5 years of age hospitalized for rotavirus diarrhea, proportion rotavirus positive and proportion vaccinated, b) Rotavirus positivity by age and rotavirus season, c) Number of rotavirus hospitalizations by age and rotavirus season, and d) distribution of diarrhea severity by season, TT Hue Province, December 2016–December 2020.

In TT Hue, the rotavirus season was slightly shifted compared to the season in Nam Dinh with annual increases in rotavirus disease from January to June and peaks less defined. In the pre-vaccine period, rotavirus positivity among children <5 years of age was lower in Hue (20.3%) than Nam Dinh (43.2%). Similar to Nam Dinh, there was a sharp decline in all-cause diarrhea hospitalizations that started in March 2020 coinciding with the start of the global COVD-19 pandemic and persisting until the end of the surveillance period (Fig. 3a).

Compared to the pre-vaccine period, no significant change in rotavirus positivity was observed overall among children <5 years of age (RR: −6.7%; 95% CI: −32.3%–14.0%) in the three years post-vaccine introduction or across more granular age groups (Fig. 3b, Table 1). Coverage with ≥1 dose of Rotavin-M1 among all children <5 years of age increased from 16.9% in the first year post-vaccine introduction to 49.5% in the third year post-vaccine introduction with the highest coverage (58.2%) achieved in the <1 year age group in the third year post-vaccine introduction. No other time period or age group achieved Rotavin-M1 coverage of 50% or greater.

The age distribution of rotavirus hospitalizations was not consistent throughout the surveillance period (Fig. 3c). In the first year post-vaccine introduction, rotavirus hospitalizations peaked in children 12–17 months of age but in the preceding and subsequent years, rotavirus hospitalizations appear more equally distributed among children 6–35 months of age. The median age of children hospitalized for rotavirus did not change significantly from 16.5 months of age (IQR: 10–27.5 months) in the pre-vaccine period compared with the first (15 months; IQR: 9–24 months; p = 0.30) or second (15.5 months; IQR: 10–25 months; p = 0.66) year post-vaccine introduction and decreased to 11 months (IQR: 8–14 months; p = 0.004) in the third year post-vaccine introduction.

Severity of diarrhea was substantially lower among children enrolled in TT Hue compared to children enrolled in Nam Dinh. Over two-thirds of children enrolled in TT Hue had mild diarrhea (Vesikari score <11). The proportion of children with moderate-to-severe diarrhea remained constant during the pre-vaccine season and first two post-vaccine introduction seasons (33.6%, 34.3%, 36.1%, respectively) and then decreased to 23.9% during the third post-vaccine season which coincided with the global COVID-19 pandemic (Fig. 3d). A similar pattern of severity was seen for children with rotavirus positive and rotavirus negative diarrhea although rotavirus positive children were substantially more likely to have moderate-to-severe diarrhea than children with rotavirus negative diarrhea (Supplemental Figure S2).

As in Nam Dinh, the predominating circulating genotypes varied from season to season in TT Hue (Fig. 2b; Supplemental Table S2). In the pre-vaccine season from January 2017–December 2017, G9P[8] (32.7%) and G3P[8] (31.8%) were the most common circulating genotypes. In the first post-vaccine season (January 2018–December 2018), G3P[8] (52.2%) was the most commonly detected genotype. In the second post-vaccine season (January 2019–December 2019), G9P[8] (20.8%) and G3P[8] (18.9%) were again the most common circulating genotypes but 18.9% of specimens could not be genotyped and another 9.4% were only partially genotyped. In the third post-vaccine season (January 2020–December 2020), G8P[8] (47.2%) was the most common genotype detected although 30.6% could either not be genotyped (13.9%) or were only partially genotyped (16.7%).

### Vaccine effectiveness

A total of 2041 children were age-eligible to be included in the vaccine effectiveness analysis and of these, 1826 (89%) had a stool specimen collected and tested and their vaccination status verified (Supplemental Figure S3). Rotavirus positive and negative children were comparable except that rotavirus positive children were older, less likely to be male, and more likely to have severe disease (Supplemental Table S3). To account for these differences and the seasonality of rotavirus disease that differed by province, all vaccine effectiveness estimates were adjusted for month and year of birth, month and year of admission, province, and sex.

**Fig. 4:**
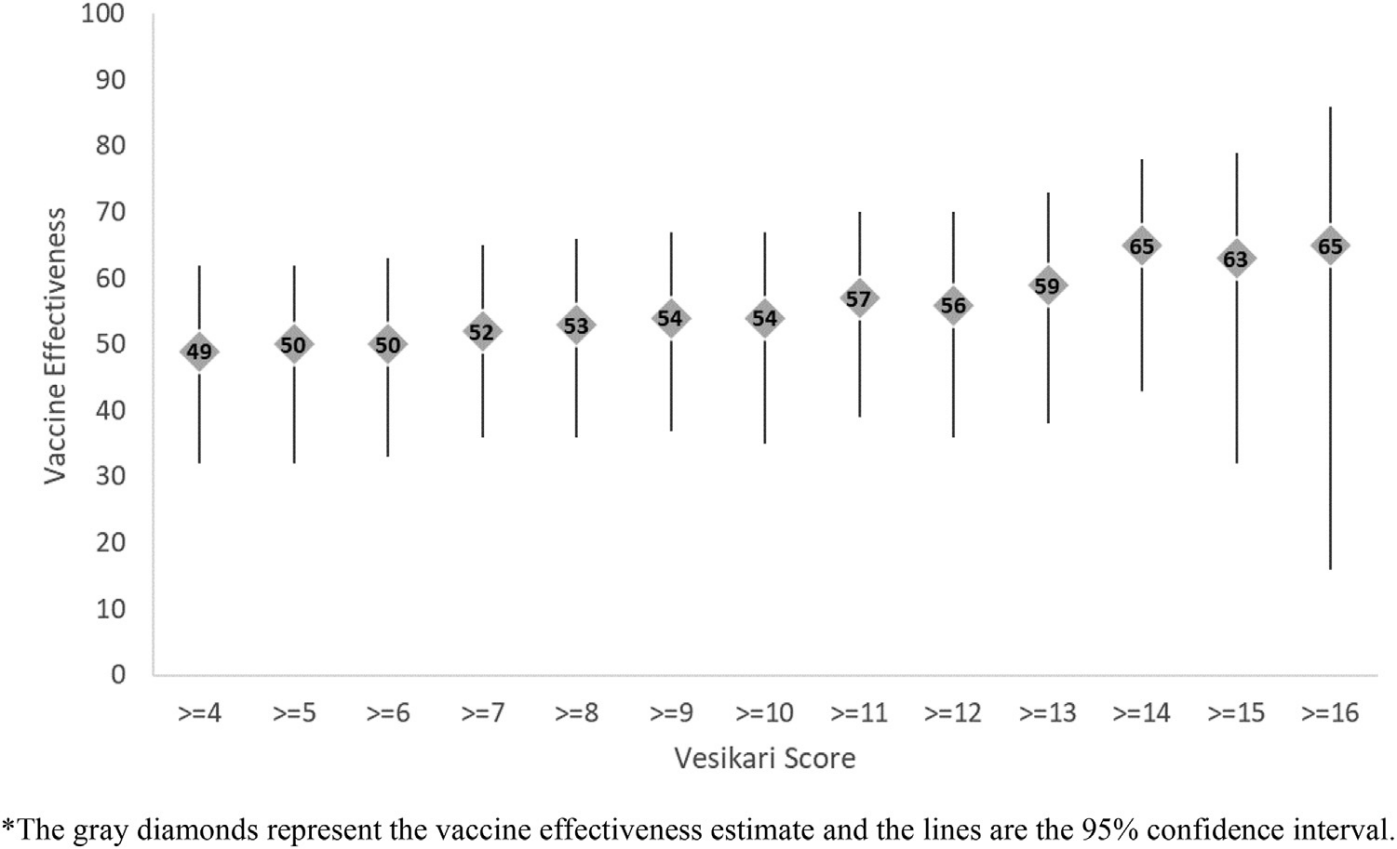
Rotavirus vaccine effectiveness and 95% confidence intervals∗ among children 6–23 months of age by Vesikari score, Nam Dinh and TT Hue Provinces, 2017–2021. ∗The gray diamonds represent the vaccine effectiveness estimate and the lines are the 95% confidence interval.

Overall, among age-eligible children 6–23 months of age, a full 2-dose series of Rotavin-M1 was 57% (95% CI: 39%, 70%) effective against hospitalization for moderate-to-severe (Vesikari score ≥11) rotavirus diarrhea and a slightly lower effectiveness of 46% (95% CI: 28%, 59%) was observed against hospitalization for any severity of rotavirus diarrhea (Table 2). Increasing effectiveness was observed with increasing severity of disease with higher effectiveness of 63% (95% CI: 32%, 79%) observed against severe disease (Vesikari score ≥15) (Fig. 4). Effectiveness against hospitalization for moderate-to-severe rotavirus diarrhea was similar in the first year of life (age 6–11 months: 52% [95% CI: 18%, 72%]) and second year (age 12–23 months: 61% [95% CI: 35%, 76%]). Vaccine effectiveness was higher in Nam Dinh than TT Hue. Among children 6–23 months of age, comparable vaccine effectiveness was observed against moderate-to-severe rotavirus diarrhea caused by the commonly circulating genotypes during the project period including G8P[8] (58% [95% CI: 25%, 77%]), G9P[8] (72% [95% CI: 23%, 90%]), G3P[8] (61% [95% CI: −17%, 87%]), and G1P[8] (94% [95% CI: 60%, 99%]).

**Table 2:** Rotavirus vaccine effectiveness against hospitalizations due to moderate-to-severe rotavirus diarrhea and rotavirus diarrhea of any severity by age, province, and circulating genotype among children 6–23 months of age, Nam Dinh and TT Hue Provinces, 2017–2021.

	Moderate-to-severe rotavirus diarrhea^a^				Any severity of rotavirus diarrhea			
	Rotavirus positive n (%)	Rotavirus negative n (%)	Crude VE (95% CI)	Adjusted^b^ VE (95% CI)	Rotavirus positive n (%)	Rotavirus negative n (%)	Crude VE (95% CI)	Adjusted^b^ VE (95% CI)
**All age-eligible children 6–23 months of age**	**n = 226**	**n = 1232**			**n = 423**	**n = 1232**		
0 doses	85 (37.6%)	333 (27.0%)	Ref	Ref	156 (36.9%)	333 (27.0%)	Ref	Ref
2 doses	141 (62.4%)	899 (73.0%)	39% (17%, 54%)	57% (39%, 70%)	267 (63.1%)	899 (73.0%)	37% (20%, 50%)	46% (28%, 59%)
**Stratified by age**								
**Children 6**–**11 months of age**	**n** = **96**	**n** = **754**			**n** = **197**	**n** = **754**		
0 doses	34 (35.4%)	223 (29.6%)	Ref	Ref	73 (37.1%)	223 (29.6%)	Ref	Ref
2 doses	62 (64.6%)	531 (70.4%)	23% (−20%, 51%)	52% (18%, 72%)	124 (62.9%)	531 (70.4%)	29% (1%, 49%)	41% (11%, 60%)
**Children 12**–**17 months of age**	**n** = **89**	**n** = **338**			**n** = **159**	**n** = **338**		
0 doses	37 (41.6%)	73 (21.6%)	Ref	Ref	58 (36.5%)	73 (21.6%)	Ref	Ref
2 doses	52 (58.4%)	265 (78.4%)	61% (36%, 76%)	71% (46%, 84%)	101 (63.5%)	265 (78.4%)	52% (27%, 68%)	55% (27%, 73%)
**Children 18**–**23 months of age**	**n** = **41**	**n** = **140**			**n** = **67**	**n** = **140**		
0 doses	14 (34.2%)	37 (26.4%)	Ref	Ref	25 (37.3%)	37 (26.4%)	Ref	Ref
2 doses	27 (65.9%)	103 (73.6%)	31% (−46%, 67%)	27% (−138%, 77%)	42 (62.7%)	103 (73.6%)	40% (−12%, 68%)	43% (−40%, 76%)
**Children 12**–**23 months of age**	**n** = **130**	**n** = **478**			**n** = **226**	**n** = **478**		
0 doses	51 (39.2%)	110 (23.0%)	Ref	Ref	83 (36.7%)	110 (23.0%)	Ref	Ref
2 doses	79 (60.8%)	368 (77.0%)	54% (30%, 69%)	61% (35%, 76%)	143 (63.3%)	368 (77.0%)	48% (27%, 63%)	49% (23%, 66%)
**Stratified by province (children 6**–**23 months of age)**								
**Nam Dinh**	**n** = **198**	**n** = **925**			**n** = **323**	**n** = **925**		
0 doses	67 (33.8%)	157 (17.0%)	Ref	Ref	95 (29.4%)	157 (17.0%)	Ref	Ref
2 doses	131 (66.2%)	768 (83.0%)	60% (44%, 72%)	65% (47%, 76%)	228 (70.6%)	768 (83.0%)	51% (34%, 64%)	55% (37%, 68%)
**TT Hue**	**n** = **28**	**n** = **307**			**n** = **100**	**n** = **307**		
0 doses	18 (64.3%)	176 (57.3%)	Ref	Ref	61 (61.0%)	176 (57.3%)	Ref	Ref
2 doses	10 (35.7%)	131 (42.7%)	25% (−67%, 67%)	31% (−70%, 72%)	39 (39.0%)	131 (42.7%)	14% (−36%, 46%)	18% (−36%, 50%)
**Stratified by genotype (children 6**–**23 months of age)**								
**G8P[8]**	**n** = **90**	**n** = **1232**			**n** = **150**	**n** = **1232**		
0 doses	29 (32.2%)	333 (27.0%)	Ref	Ref	49 (32.7%)	333 (27.0%)	Ref	Ref
2 doses	61 (67.8%)	899 (73.0%)	22% (−23%, 51%)	58% (25%, 77%)	101 (67.3%)	899 (73.0%)	24% (−10%, 47%)	48% (17%, 67%)
**G9P[8]**	**n** = **23**	**n** = **1232**			**n** = **42**	**n** = **1232**		
0 doses	14 (60.9%)	333 (27.0%)	Ref	Ref	20 (47.6%)	333 (27.0%)	Ref	Ref
2 doses	9 (39.1%)	899 (73.0%)	76% (46%, 90%)	72% (23%, 90%)	22 (52.4%)	899 (73.0%)	59% (24%, 78%)	52% (−2%, 78%)
**G3P[8]**	**n** = **21**	**n** = **1232**			**n** = **56**	**n** = **1232**		
0 doses	11 (52.4%)	333 (27.0%)	Ref	Ref	29 (51.8%)	333 (27.0%)	Ref	Ref
2 doses	10 (47.6%)	899 (73.0%)	66% (20%, 86%)	61% (−17%, 87%)	27 (48.2%)	899 (73.0%)	65% (41%, 80%)	46% (−5%, 72%)
**G1P[8]**	**n** = **16**	**n** = **1232**			**n** = **24**	**n** = **1232**		
0 doses	7 (43.8%)	333 (27.0%)	Ref	Ref	11 (45.8%)	333 (27.0%)	Ref	Ref
2 doses	9 (56.3%)	899 (73.0%)	52% (−29%, 82%)	94% (60%, 99%)	13 (54.2%)	899 (73.0%)	56% (1%, 81%)	77% (28%, 93%)

a Moderate-to-severe diarrhea defined as a Vesikari score ≥11.

b Vaccine effectiveness estimates adjusted for month and year of birth, month and year of admission, province, and sex.

## Discussion

A full 2-dose series of Rotavin-M1 was 57% effective against hospitalization for moderate-to-severe rotavirus diarrhea among children 6–23 months of age in two provinces of Vietnam. As has been seen with other rotavirus vaccines, effectiveness increased with increasing severity of disease^16^ and the vaccine provided protection against the multiple different genotypes circulating during the surveillance period.^17^ Protection extended into the second year of life although fewer children 18–23 months of age were enrolled compared with children 6–17 months of age, which largely reflects the epidemiology of rotavirus disease in Vietnam. As most children who initiated the two-dose vaccine series completed it, we were unable to calculate the vaccine effectiveness for children who received only one dose of vaccine. The full-series vaccine effectiveness estimate is similar to the efficacy (63.9%; 95% CI: 7.6%, 90.9%) of RotaTeq vaccine against moderate-to-severe rotavirus diarrhea during the first 2 years of life from a clinical trial that was conducted in urban and peri-urban Nha Trang, Vietnam.^18^ Rotavin-M1 was licensed in Vietnam based on immunogenicity data so no efficacy estimate is available from the initial clinical trials for this vaccine. Our estimate is slightly lower but similar to rotavirus vaccine effectiveness (69.7%; 95% CI: 53.3%, 80.6%) from a post-licensure evaluation of rotavirus vaccines administered on the private market in Ho Chi Minh with almost two thirds of vaccinated children having received Rotarix vaccine.^19^ In our evaluation, Rotavin-M1 was administered through the routine expanded program on immunization system with all children living in the selected districts eligible to receive the vaccine.

Hospitalizations due to rotavirus diarrhea decreased in Nam Dinh following vaccine introduction. Rotavirus disease in this province had sharp seasonal peaks that were blunted following Rotavin-M1 introduction. As age-restrictions were used to administer the vaccine, children who presented late (at ≥14 weeks of age) to initiate their primary infant vaccine series were not eligible to receive Rotavin-M1 vaccine. Thus, population coverage increased more slowly and peaked at 70–75% during the third post-vaccine introduction year. This level of coverage left a pool of susceptible children that accumulated during the second year post-vaccine introduction and likely enabled increased circulation of the virus in the population. However, while the proportion of children who tested positive during the second post-vaccine year increased from the first post-vaccine introduction year, rotavirus positivity remained below the pre-vaccine introduction level. This biennial pattern of increased rotavirus activity has also been observed in the United States where age-restrictions remain in place and rotavirus vaccination coverage has plateaued at approximately 70%.^20^ In TT Hue, given the substantially lower rotavirus vaccination coverage and the lower severity of rotavirus hospitalizations, decreases in rotavirus hospitalizations among children <5 years of age following rotavirus vaccine introduction were less well pronounced compared with Nam Dinh, and population level trends were difficult to discern. Similarly, the smaller sample size and less severe disease among hospitalized children in TT Hue resulted in lower vaccine effectiveness with wide confidence intervals crossing the null compared to higher, more precise estimates in Nam Dinh which had a larger sample size and children with more severe rotavirus disease. Rotavirus vaccines are more effective against severe rotavirus disease and countries that quickly achieved and maintained high coverage for rotavirus vaccination have seen sustained reductions in severe rotavirus disease burden.^6^^,^^7^

Our evaluation had several limitations. First, we introduced rotavirus vaccine in during the height of the rotavirus season in Nam Dinh. Thus, our pre-vaccine period included a few months where the youngest children would have been eligible for rotavirus vaccine. This may have resulted in an underestimate of the vaccine impact in subsequent years. However, given the epidemiology of rotavirus disease in Vietnam, rotavirus infections in the youngest age groups are uncommon and therefore may have had minimal impact on our estimates of reduction. Central Hue Hospital stopped enrolling early in the post-vaccine period and was excluded from trend analyses which reduced the number and severity of disease of children included in this analysis as Central Hue Hospital is a tertiary referral hospital. Furthermore, vaccination coverage was lower in TT Hue province. Given the timing of vaccine introduction relative to the first post-vaccine introduction season in TT Hue, the low coverage and corresponding low impact during this first season may be partially attributable to the scaling up of the vaccination program. The differences in coverage between the two provinces may also be due to regional cultural differences. Parents in TT Hue often avoid exposure to needles for their children during the first couple months of life and therefore present later to vaccination clinic to initiate the infant vaccine series after children have aged out of the window of eligibility for rotavirus vaccine. Taken together, the smaller sample size and lower coverage likely made population level trends difficult to observed in TT Hue. In the later years of surveillance, more milder cases were enrolled and the likely lower viral load in these cases made genotyping difficult and resulted in an increased proportion of specimens which could not be genotyped. Because most children who started the rotavirus vaccine series completed it and rotavirus vaccination does not prevent other causes of diarrhea, we used the proportion of children hospitalized for non-rotavirus diarrhea who had received at least one dose of rotavirus vaccine as a proxy for rotavirus vaccine coverage in the population. We recognize that this is a crude proxy for vaccine coverage in the population and further analysis of overall coverage in the population is warranted. If children had differential access to hospitalization for diarrhea than for immunizations, we may have under- or over-estimated vaccine coverage in the population. Furthermore, we only had three years of post-vaccine introduction surveillance and thus, not all children <5 years of age were eligible to receive the vaccine. Monitoring the long-term impact of rotavirus vaccination programs after the under five years population has stabilized at full vaccine coverage is important. Finally, the global COVID-19 pandemic began in the third year post-vaccine introduction and likely affected care-seeking behavior during this period. Overall admissions for diarrhea notably decreased in both provinces and children that were admitted had notably less severe diarrhea than in previous years. We are unable to disentangle to effect of the pandemic on care-seeking behavior and the impact of the vaccine on rotavirus disease burden during this period.

In summary, as rotavirus vaccination coverage increased, we observed substantial declines in rotavirus hospitalizations following Rotavin-M1 introduction in Nam Dinh with robust vaccine effectiveness observed into the second year of life and against the predominant circulating strains. Vaccine impact was harder to discern at the population level in TT Hue where vaccination coverage is lower. Rollout of this vaccine at a national level with efforts to achieve high coverage in all districts may have substantial impact on the rotavirus disease burden in Vietnam.

## Contributors

Nguyen Van Trang, Jacqueline E. Tate, Umesh D. Parashar and Dang Duc Anh were responsible for study design, data analysis, study monitor and manuscript preparation. Duong Nhu Tran, Nguyen Dang Hien, Huong Thuy Nguyen, Le Thi Phuong Mai, Thiem Dinh Vu and Quyet Tu Nguyen were responsible for oversight field activities for both study sites. The Members of the Rotavin VE team were responsible for carrying out vaccination and diarrhea surveillance in the two study sites. Le Thi Khanh Ly, Mai Ngoc Thi Chu, Mai Phuong Ngoc Tran and Thao Phuong Thi Pham were responsible for laboratory analysis of the samples, data entry and cleaning, safety monitoring. Catherine Yen and Baoming Jiang were responsible for protocol development and study design. All authors were involved in manuscript review.

## Data sharing statement

Some data were included in the PhD dissertation of Thao Phuong Thi Pham, to be defended by December 2022 at the Center for Training and Research Management, NIHE, Vietnam.

## Disclaimer

The findings and conclusions in this report are those of the authors and do not necessarily represent the official position of the Centers for Disease Control and Prevention.

## Declaration of interests

Huong Thuy Nguyen, Thao Phuong Thi Pham, Nguyen Dang Hien are the member of POLYVAC-Vietnam.
