# Supplementary material for: Impact and effectiveness of Rotavin-M1 under conditions of routine use in two provinces in Vietnam, 2016–2021, an observational and case–control study
Source: Lancet Reg Health West Pac. 2023 May 18;37:100789. doi: 10.1016/j.lanwpc.2023.100789 (PMC10485664; doi:10.1016/j.lanwpc.2023.100789)
Supplement: Supplemental Table S1–S3 [file mmc1.docx]

Supplemental Table 1. Enrollment, specimen collection, rotavirus testing, and results for children <5 years of age hospitalized for diarrhea at participating sentinel surveillance sites, and Rotavin-M1 coverage in age-eligible children, Nam Dinh and TT Hue Provinces, 2017-2021

|  | **Nam Dinh** | **TT Hue** | **Total** |
| --- | --- | --- | --- |
| Enrolled children | 4,662 | 2,566 | 7,228 |
| Stool specimen collected | 4,428 (95%) | 2,349 (92%) | 6,777 (94%) |
| Specimens tested by ELISA | 4,366 (99%) | 2,260 (96%) | 6,626 (98%) |
| Rotavirus positive | 1,553 (35%) | 611 (26%) | 2,164 (32%) |
| Age-eligible to receive rotavirus vaccine* | 1,377 | 489 | 1,866 |
| Received at least one dose of Rotavin-M1 | 1,066 (77%) | 203 (42%) | 1,269 (68%) |

*Restricted to children that were hospitalized for non-rotavirus diarrhea and had a verified vaccination status.

Supplemental Table 2. Distribution of circulating genotypes by province and season, Nam Dinh and TT Hue, December 2016-March 2021

|  | **Apr 2017-Mar 2018** | | **Apr 2018-Mar 2019** | | | **Apr 2019-Mar 2020** | | **Apr 2020-Mar 2021** | | |
| --- | --- | --- | --- | --- | --- | --- | --- | --- | --- | --- |
| **Nam Dinh** | **n=357** | **%** | **N=188** | **%** | | **N=320** | **%** | **N=80** | **%** | |
| G1P[8] | 0 | 0.0 | 4 | 2.1 | | 3 | 0.9 | 19 | 23.8 | |
| G3P[8] | 16 | 4.5 | 37 | 19.7 | | 19 | 5.9 | 6 | 7.5 | |
| G8P[8] | 4 | 1.1 | 11 | 5.9 | | 215 | 67.2 | 15 | 18.8 | |
| G9P[8] | 267 | 74.8 | 91 | 48.4 | | 22 | 6.9 | 0 | 0.0 | |
| Mixed | 27 | 7.6 | 8 | 4.3 | | 5 | 1.6 | 2 | 2.5 | |
| Partial G | 3 | 0.8 | 2 | 1.1 | | 10 | 3.1 | 6 | 7.5 | |
| Partial P | 6 | 1.7 | 6 | 3.2 | | 10 | 3.1 | 7 | 8.8 | |
| Non-typeable | 5 | 1.4 | 12 | 6.4 | | 24 | 7.5 | 21 | 26.3 | |
| Other | 29 | 8.1 | 17 | 9.0 | | 12 | 3.8 | 4 | 5.0 | |
|  | **Jan 2017-Dec 2017** | | **Jan 2018-Dec 2018** | | | **Jan 2019-Dec 2019** | | **Jan 2020-Dec 2020** | | |
| **TT Hue** | **n=107** | **%** | **n=205** | | **%** | **n=53** | **%** | **n=36** | | **%** |
| G1P[8] | 0 | 0.0 | 1 | | 0.5 | 3 | 5.7 | 3 | | 8.3 |
| G2P[4] | 4 | 3.7 | 20 | | 9.8 | 0 | 0.0 | 0 | | 0.0 |
| G3P[8] | 34 | 31.8 | 107 | | 52.2 | 10 | 18.9 | 4 | | 11.1 |
| G8P[8] | 15 | 14.0 | 0 | | 0.0 | 8 | 15.1 | 17 | | 47.2 |
| G9P[8] | 35 | 32.7 | 22 | | 10.7 | 11 | 20.8 | 1 | | 2.8 |
| Mixed | 5 | 4.7 | 11 | | 5.4 | 4 | 7.5 | 0 | | 0.0 |
| Partial G | 2 | 1.9 | 6 | | 2.9 | 1 | 1.9 | 6 | | 16.7 |
| Partial P | 2 | 1.9 | 7 | | 3.4 | 4 | 7.5 | 0 | | 0.0 |
| Non-typeable | 5 | 4.7 | 22 | | 10.7 | 10 | 18.9 | 5 | | 13.9 |
| Other | 5 | 4.7 | 9 | | 4.4 | 2 | 3.8 | 0 | | 0.0 |

Supplemental Table 3. Sociodemographic and clinical characteristics of children included in the vaccine effectiveness analysis stratified by rotavirus positivity and severity of rotavirus disease, Nam Dinh and TT Hue Provinces, 2017-2021

|  | **Rotavirus negative** | **Rotavirus positive** | | | |
| --- | --- | --- | --- | --- | --- |
|  |  | **Moderate-to Severe** | **p-value** | **Any severity** | **p-value** |
| **Sociodemographic Characteristics** | **n/N (%)** | **n/N (%)** |  | **n/N (%)** |  |
| **Male** | 785/1232 (63.7%) | 125/226 (55.3%) | 0.02 | 234/423 (55.3%) | 0.002 |
|  | 10 (7-14) | 12 (9-16) | <0.001 | 12 (8-15) | <0.001 |
| **Age groups** |  |  | <0.001 |  | <0.001 |
| 6-8 months | 439/1232 (35.6%) | 51/226 (22.6%) |  | 112/423 (26.5%) |  |
| 9-11 months | 315/1232 (25.6%) | 45/226 (19.9%) |  | 85/423 (20.1%) |  |
| 12-17 months | 338/1232 (27.4%) | 89/226 (39.4%) |  | 159/423 (37.6%) |  |
| 18-23 months | 140/1232 (11.4%) | 41/226 (18.1%) |  | 67/423 (15.8%) |  |
| **Chronic medical condition** | 549/1228 (44.7%) | 124/226 (54.9%) | 0.005 | 200/422 (47.4%) | 0.34 |
| **Mother’s median age in years (IQR)** | 28 (25-32) | 28 (25-31) | 0.82 | 28 (25-32) | 0.32 |
| **Mother’s highest education level** |  |  | 0.23 |  | 0.51 |
| None | 9/1232 (0.7%) | 1/226 (0.4%) |  | 1/423 (0.2%) |  |
| Primary | 59/1232 (4.8%) | 5/226 (2.2%) |  | 19/423 (4.5%) |  |
| Secondary | 546/1232 (44.3%) | 95/226 (42.0%) |  | 187/423 (44.2%) |  |
| Post-Secondary | 440/1232 (35.7%) | 83/226 (36.7%) |  | 143/423 (33.8%) |  |
| University or above | 178/1232 (14.4%) | 42/226 (18.6%) |  | 73/423 (17.3%) |  |
| **Mother’s marital status** |  |  | 0.73 |  | 0.51 |
| Married | 1204/1232 (97.7%) | 219/226 (96.9%) |  | 410/423 (96.9%) |  |
| Single | 6/1232 (0.5%) | 2/226 (0.9%) |  | 2/423 (0.5%) |  |
| Divorced | 2/1232 (0.2%) | 0 /226 (0.0%) |  | 0 /423 (0.0%) |  |
| Co-habitation | 20/1232 (1.6%) | 5/226 (2.2%) |  | 11/423 (2.6%) |  |
| **Father’s highest education level** |  |  | 0.53 |  | 0.18 |
| None | 11/1231 (0.9%) | 1/226 (0.4%) |  | 1/423 (0.2%) |  |
| Primary | 56/1231 (4.6%) | 8/226 (3.5%) |  | 20/423 (4.7%) |  |
| Secondary | 532/1231 (43.2%) | 111/226 (49.1%) |  | 208/423 (49.2%) |  |
| Post-Secondary | 459/1231 (37.3%) | 78/226 (34.5%) |  | 140/423 (33.1%) |  |
| University or above | 173/1231 (14.1%) | 28/226 (12.4%) |  | 54/423 (12.8%) |  |
| **Median number of people in household (IQR)** | 5 (4-5) | 5 (4-6) | 0.33 | 5 (4-6) | 0.10 |
| **Median number of children in household (IQR)** | 1 (1-2) | 1 (1-2) | 0.10 | 1 (1-2) | 0.11 |
| **Source of drinking water** |  |  | 0.03 |  | 0.85 |
| Tap to house | 703/1232 (57.1%) | 110/226 (48.7%) |  | 247/423 (58.4%) |  |
| Bore hole | 456/1232 (37.0%) | 95/226 (42.0%) |  | 150/423 (35.5%) |  |
| Other | 73/1232 (5.9%) | 21/226 (9.3%) |  | 26/423 (6.3%) |  |
| **Year of Enrollment** |  |  | <0.001 |  | <0.001 |
| 2018 | 294/1232 (23.9%) | 26/226 (11.5%) |  | 60/423 (14.2%) |  |
| 2019 | 528/1232 (42.9%) | 130/226 (57.5%) |  | 217/423 (51.3%) |  |
| 2020 | 314/1232 (25.5%) | 43/226 (19.0%) |  | 103/423 (24.4%) |  |
| 2021 | 96/1232 (7.8%) | 27/226 (12.0%) |  | 43/423 (10.2%) |  |
| **Month of Enrollment** |  |  | <0.001 |  | <0.001 |
| January | 54/1232 (4.4%) | 26/226 (11.5%) |  | 50/423 (11.8%) |  |
| February | 51/1232 (4.1%) | 18/226 (8.0%) |  | 43/423 (10.2%) |  |
| March | 64/1232 (5.2%) | 20/226 (8.9%) |  | 35/423 (8.3%) |  |
| April | 88/1232 (7.1%) | 16/226 (12.0%) |  | 22/423 (5.2%) |  |
| May | 87/1232 (7.1%) | 20/226 (8.9%) |  | 42/423 (9.9%) |  |
| June | 103/1232 (8.4%) | 10/226 (4.4%) |  | 21/423 (5.0%) |  |
| July | 134/1232 (10.9%) | 8/226 (3.5%) |  | 19/423 (4.5%) |  |
| August | 127/1232 (10.3%) | 9/226 (4.0%) |  | 23/423 (5.4%) |  |
| September | 158/1232 (12.8%) | 15/226 (6.6%) |  | 26/423 (6.2%) |  |
| October | 151/1232 (12.3%) | 14/226 (6.2%) |  | 26/423 (6.2%) |  |
| November | 114/1232 (9.3%) | 23/226 (10.2%) |  | 36/423 (8.5%) |  |
| December | 101/1232 (8.2%) | 47/226 (20.8%) |  | 80/423 (18.9%) |  |
| **Province** |  |  | <0.001 |  | 0.60 |
| Nam Dinh | 925/1232 (75.1%) | 198/226 (87.6%) |  | 323/423 (76.4%) |  |
| TT Hue | 307/1232 (24.9%) | 28/226 (12.4%) |  | 100/423 (23.6%) |  |
| **Clinical Characteristics** |  |  |  |  |  |
| **Median duration of diarrhea in days (IQR)** | 2 (1-3) | 2 (1-2) | 0.10 | 2 (1-2) | 0.03 |
| **Duration of Diarrhea** |  |  | 0.20 |  | 0.12 |
| 0-4 days | 1165/1232 (94.6%) | 219/226 (96.9%) |  | 407/423 (96.2%) |  |
| 5 days | 22/1232 (1.8%) | 4/226 (1.8%) |  | 9/423 (2.1%) |  |
| ≥6 days | 45/1232 (3.7%) | 3/226 (1.3%) |  | 7/423 (1.7%) |  |
| **Median max number of diarrhea episodes in 24 hrs (IQR)** | 5 (4-7) | 6 (5-8) | <0.001 | 5 (4-7) | 0.003 |
| **Maximum number of diarrhea episodes in 24 hrs** |  |  | <0.001 |  | 0.004 |
| 1-3 episodes | 168/1229 (13.7%) | 15/226 (6.6%) |  | 40/423 (9.5%) |  |
| 4-5 episodes | 552/1229 (44.9%) | 85/226 (37.6%) |  | 172/423 (40.7%) |  |
| ≥6 episodes | 509/1229 (41.4%) | 126/226 (55.8%) |  | 211/423 (49.9%) |  |
| **Has Vomiting** | 794/1232 (64.5%) | 219/226 (96.9%) | <0.001 | 293/423 (69.0%) | 0.09 |
| **If vomiting, median duration of vomiting in days (IQR)** | 2 (1-3) | 2 (1-2) | 0.92 | 2 (1-2) | 0.13 |
| **If vomiting, duration of vomiting in days** |  |  | 0.92 |  | 0.24 |
| 1 day | 221/788 (28.1%) | 61/219 (27.9%) |  | 93/292 (31.9%) |  |
| 2 days | 367/788 (46.6%) | 105/219 (48.0%) |  | 138/292 (47.3%) |  |
| ≥3 days | 200/788 (25.4%) | 53/219 (24.2%) |  | 61/292 (20.9%) |  |
| **If vomiting, median max no. vomiting episodes in 24 hrs (IQR)** | 4 (3-5) | 4 (3-5) | 0.11 | 4 (3-5) | 0.41 |
| **If vomiting, maximum number of vomiting episodes in 24 hrs** |  |  | 0.25 |  | 0.73 |
| 1 episode | 21/780 (2.7%) | 3/219 (1.4%) |  | 6/288 (2.1%) |  |
| 2-4 episodes | 464/780 (59.5%) | 122/219 (55.7%) |  | 167/288 (58.0%) |  |
| ≥5 episodes | 295/780 (37.8%) | 94/219 (42.9%) |  | 115/288 (39.9%) |  |
| **History of fever** | 814/1232 (66.1%) | 185/226 (81.9%) | <0.001 | 289/423 (68.3%) | 0.40 |
| **Maximum recorded temperature** |  |  | 0.63 |  | 0.18 |
| 35^◦^-37^◦^ C | 144/1003 (14.4%) | 32/226 (14.2%) |  | 61/366 (16.7%) |  |
| >37^◦^-<38.5^◦^ C | 445/1003 (44.4%) | 93/226 (41.2%) |  | 142/366 (38.8%) |  |
| ≥38.5^◦^-<39^◦^ C | 177/1003 (17.7%) | 48/226 (21.2%) |  | 78/366 (21.3%) |  |
| ≥39^◦^C | 237/1003 (23.6%) | 53/226 (23.5%) |  | 85/366 (23.2%) |  |
| **Received ORS before admission** | 231/1208 (19.1%) | 34/223 (15.3%) | 0.17 | 69/414 (16.7%) | 0.27 |
| **Received ORS after admission** | 1176/1231 (95.5%) | 219/226 (96.9%) | 0.35 | 408/423 (96.5%) | 0.42 |
| **Child’s general condition** |  |  | <0.001 |  | 0.04 |
| Well, alert | 956/1231 (77.7%) | 128/226 (56.6%) |  | 306/423 (72.3%) |  |
| Restless, irritable | 272/1231 (22.1%) | 98/226 (43.4%) |  | 117/423 (27.7%) |  |
| Lethargic or unconscious | 3/1231 (0.2%) | 0/226 (0.0%) |  | 0/423 (0.0%) |  |
| **Sunken eyes** | 442/1232 (35.9%) | 135/226 (59.7%) | 0.001 | 176/421 (59.7%) | 0.03 |
| **Child’s thirst status** |  |  | <0.001 |  | 0.04 |
| Drank normally, not thirsty | 588/1232 (47.7%) | 60/226 (26.6%) |  | 176/423 (41.6%) |  |
| Thirsty, drank eagerly | 640/1232 (52.0%) | 166/226 (72.5%) |  | 247/423 (58.4%) |  |
| Drank poorly or not able to drink | 4/1232 (0.3%) | 0/226 (0.0%) |  | 0/423 (0.0%) |  |
| **Skin Turgor** |  |  | <0.001 |  | 0.03 |
| Goes back quickly (i.e. immediately) | 778/1219 (63.8%) | 93/226 (41.2%) |  | 244/420 (58.1%) |  |
| Goes back slowly (i.e. 1-2 seconds) | 430/1219 (35.3%) | 133/226 (58.9%) |  | 175/420 (41.7%) |  |
| Goes back very slowly (i.e. >2 seconds) | 11/1219 (0.9%) | 0/226 (0.0%) |  | 1/420 (0.2%) |  |
| **Dehydration Status** |  |  | <0.001 |  | 0.10 |
| None | 697/1232 (56.6%) | 76/226 (33.6%) |  | 220/423 (52.0%) |  |
| Mild | 530/1232 (43.0%) | 150/226 (66.4%) |  | 203/423 (48.0%) |  |
| Severe | 5/1232 (0.4%) | 0/226 (0.0%) |  | 0/423 (0.0%) |  |
| **Given IV Fluids** | 797/1231 (64.7%) | 203/226 (89.8%) | <0.001 | 310/422 (73.5%) | 0.001 |
| **Median Vesikari Score (IQR)** | 11 (9-14) | 13 (12-15) | <0.001 | 12 (9-14) | 0.03 |
