# Supplementary material for: Impact and effectiveness of Rotavin-M1 under conditions of routine use in two provinces in Vietnam, 2016–2021, an observational and case–control study
Source: Lancet Reg Health West Pac. 2023 May 18;37:100789. doi: 10.1016/j.lanwpc.2023.100789 (PMC10485664; doi:10.1016/j.lanwpc.2023.100789)

Supplemental Figure 2. Distribution of rotavirus severity by season among children <5 years of age with a) rotavirus positive diarrhea and b) rotavirus negative diarrhea, TT Hue Province, January 2017-December 2020

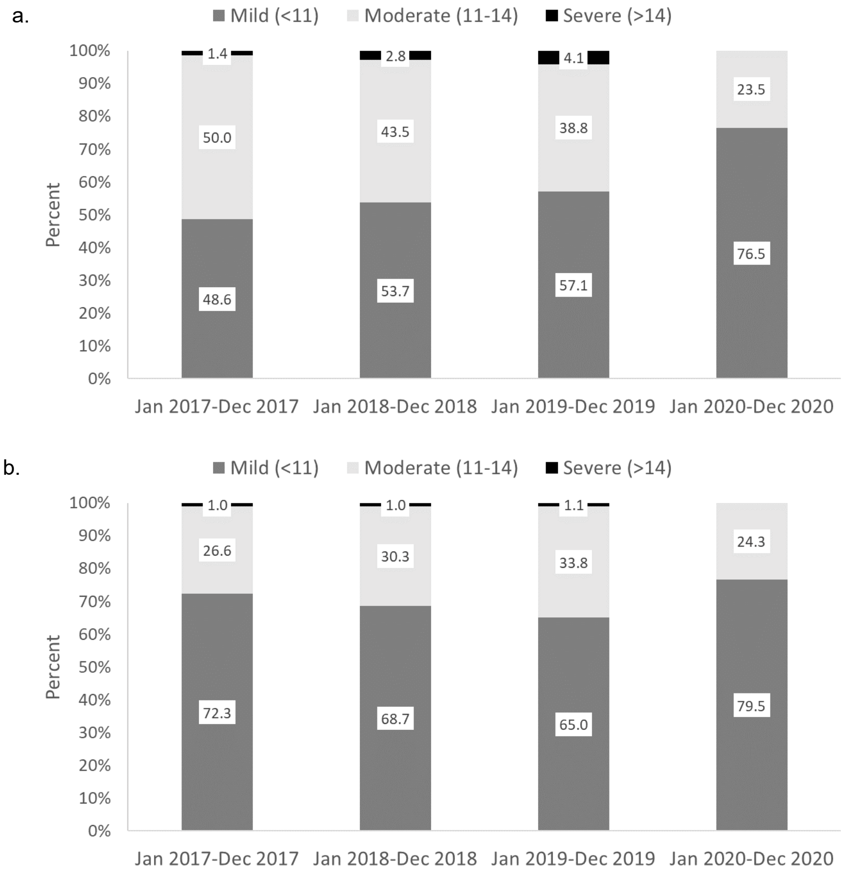

Supplement: Supplemental Figure S2 [file mmc3.pdf]
